# Artificial association of memory events by optogenetic stimulation of hippocampal CA3 cell ensembles

**DOI:** 10.1186/s13041-018-0424-1

**Published:** 2019-01-08

**Authors:** Naoya Oishi, Masanori Nomoto, Noriaki Ohkawa, Yoshito Saitoh, Yoshitake Sano, Shuhei Tsujimura, Hirofumi Nishizono, Mina Matsuo, Shin-ichi Muramatsu, Kaoru Inokuchi

**Affiliations:** 1 Department of Biochemistry, Graduate School of Medicine and Pharmaceutical Sciences, University of Toyama, Toyama, 930-0194 Japan; 2 Core Research for Evolutional Science and Technology (CREST), Japan Science and Technology Agency (JST), University of Toyama, Toyama, 930-0194 Japan; 3 Precursory Research for Embryonic Science and Technology (PRESTO), JST, University of Toyama, Toyama, 930-0194 Japan; 4 Division of Animal Experimental Laboratory, Life Science Research Center, University of Toyama, Toyama, 930-0194 Japan; 5 Division of Neurology, Department of Medicine, Jichi Medical University, Tochigi, 329-0498 Japan; 6 Center for Gene and Cell Therapy, The Institute of Medical Science, The University of Tokyo, Tokyo, 108-8639 Japan; 7 Present address: Department of Applied Biological Science, Faculty of Science and Technology, Tokyo University of Science, Noda, Chiba, 278-8510 Japan

**Keywords:** Hippocampus, CA3, Recurrent circuit, Artificial association, Synaptic plasticity, Long-term potentiation (LTP), Optogenetics

## Abstract

Previous gain-of-function studies using an optogenetic technique showed that manipulation of the hippocampal dentate gyrus or CA1 cell ensembles is important for memory reactivation and to generate synthetic or false memory. However, gain-of-function study manipulating CA3 cell ensembles has not been reported. The CA3 area of the hippocampus comprises a recurrent excitatory circuit, which is thought to be important for the generation of associations among the stored information within one brain region. We investigated whether the coincident firing of cell ensembles in one brain region, hippocampal CA3, associates distinct events. CA3 cell ensembles responding to context exploration and during contextual fear conditioning were labeled with channelrhodopsin-2 (ChR2)-mCherry. The synchronous activation of these ensembles induced freezing behavior in mice in a neutral context, in which a foot shock had never been delivered. The recall of this artificial associative fear memory was context specific. In vivo electrophysiological recordings showed that 20-Hz optical stimulation of ChR2-mCherry-expressing CA3 neurons, which is the same stimulation protocol used in behavioral experiment, induced long-term potentiation at CA3-CA3 synapses. Altogether, these results demonstrate that the synchronous activation of ensembles in one brain region, CA3 of the hippocampus, is sufficient for the association of distinct events. The results of our electrophysiology potentially suggest that this artificial association of memory events might be induced by the strengthening of synaptic efficacy between CA3 ensembles via recurrent circuit.

**Electronic supplementary material:**

The online version of this article (10.1186/s13041-018-0424-1) contains supplementary material, which is available to authorized users.

## Introduction

Subpopulation of neurons that were activated during learning, is reactivated during retrieval [1–6] and that activation of the specific neuronal ensemble is required and sufficient to retrieve that memory [7–10]. These findings indicate that memories are stored in cell ensemble activated during learning.

Acquisition of a new memory is susceptible to modification by the simultaneous and artificial activation of a specific neural ensemble corresponding to that pre-stored memory, generating synthetic or false memories [4, 11]. Retrieval of two independent memories by natural cue or optogenetic stimulation associates distinct events [12, 13].

Coincident activation of neurons results in a strengthening in synaptic efficacy such as long-term potentiation (LTP) [14, 15]. LTP at appropriate synapses are both necessary and sufficient for information storage [16, 17], and it also contributes to associative learning or memory update [18–20].

Previous gain-of-function studies using an optogenetic technique showed that manipulation of the hippocampal dentate gyrus (DG) [4, 8, 21–24] or CA1 [12] cell ensembles is important for memory reactivation and to generate synthetic or false memory by linking between stored information and sensory input by artificial activation of cell ensembles. However, gain-of-function study manipulating hippocampal CA3 cell ensembles has not been reported.

Pyramidal cells in the CA3 region of the hippocampus make synapses with each other via recurrent collaterals [25, 26]. This recurrent excitatory circuit has a key role in retrieving whole pattern from degraded cue, a process called pattern completion [27–29]. The hippocampal CA3 is reported to have an important role in the associative learning [30, 31], and this recurrent network is thought to form the associative memory within one brain region [30]. However, there is no experimental evidence demonstrating that CA3 region is important for the incorporation of previously stored information within one brain region to generate associative memories.

We hypothesized that the coincident firing of cell ensembles of the hippocampal CA3, which have recurrent network within one brain region, integrates distinct events. Also, we tested whether the synchronous activation of CA3 induces LTP in CA3-CA3 synapses. Here we showed that the synchronous activation of ensembles in CA3 associates distinct events. Also, in vivo electrophysiological recording showed that 20-Hz optical stimulation of ChR2-mCherry expressing CA3 neurons, which is the same stimulation protocol used in behavioral experiment, induces LTP in CA3-CA3 synapses. This results of electrophysiology potentially suggest that the artificial association of memory events might be induced by the strengthening of synaptic efficacy between CA3 ensembles via recurrent circuit.

## Methods

### Animals and genotyping

The c-fos::tetracycline transactivator (tTA) mice were purchased from the Mutant Mouse Regional Resource Center (stock no. 031756-MU). The KA1::Cre mice were purchased from Jackson Laboratory (Jackson Laboratory stock no: 006474, G32–4 Cre). Floxed-NR1 mice (Jackson Laboratory stock no: 005246) were donated by Drs. S. Tonegawa (RIKEN-Massachusetts Institute of Technology) and S. Itohara (RIKEN Brain Science Institute).

The c-fos::tTA/KA1::Cre double transgenic mice for behavioral analyses were generated via in vitro fertilization with eggs from C57BL/6 J mice and embryo transfer techniques [12]. The CA3 pyramidal cell-restricted *N*-methyl-D-aspartate (NMDA) receptor knock-out (CA3-NR1 KO) mice were generated from KA1::Cre mice and homozygous floxed-NR1 mice via in vitro fertilization.

The c-fos::tTA mice were backcrossed with C57BL/6 J at least 10 times after we purchased from the Mutant Mouse Regional Resource Center. The KA1::Cre mice was mixed C57BL/6 J and C57BL/6 N genetic background when we purchased from Jackson Laboratory. The results of A 32 SNP (single nucleotide polymorphism) panel analysis, which conducted by Jackson Laboratory showed that, at least 3 of 5 markers that distinguish C57BL/6 J from C57BL/6 N were found to be C57BL/6 J type [32]. After we obtained the KA1::Cre mice, the mice were backcrossed with C57BL/6 J at least 5 times to be C57BL/6 J background. The fNR1 mice were backcrossed with C57BL/6 J more than 25 times. Therefore, all experimental mice were C57BL/6 J background. Male mice were used for all the experiment.

The mice were maintained on a 12-h light-dark cycle (lights on 8:00 am) at 24 ± 3 °C and 55 ± 5% humidity with food and water ad libitum and were housed with littermates until the surgeries. All procedures involving the use of animals complied with the guidelines of the National Institutes of Health and were approved by the Animal Care and Use Committee of the University of Toyama.

c-fos::tTA mice were genotyped via polymerase chain reaction (PCR) with genomic DNA isolated from the tails of the pups as described previously [33]. To detect the Cre recombinase transgene in KA1::Cre mice, 5′-ACCTGATGGACATGTTCAGGGATCG-3′ and 5′-TCCGGTTATTCAACTTGCACCATGC-3′ primers were used with PCR conditions of 94 °C for 2 min, 35 cycles of 94 °C for 30 s, 55 °C for 15 s, and 72 °C for 20 s, followed by 72 °C for 7 min. For floxed-NR1 mice, the primers 5′-GCTTGGGTGGAGAGGCTATTC-3′ and 5′-CAAGGTGAGATGACAGGAGATC-3′ to detect the neomycin resistance cassette and 5′-TGTGCTGGGTGTGAGGGTTG-3′ and 5′-GTGAGCTGCACTTCCAGAAG-3′ to detect the NR1 locus were used with PCR conditions of 94 °C for 2 min, 30 cycles of 94 °C for 30 s, 60 °C for 60 s, and 72 °C for 30 s, followed by 72 °C for 7 min.

### Viral vectors

The pAAV-EF1α::DIO-ChR2(T159C)-mCherry plasmid was donated by Dr. K. Deisseroth, Stanford University. The pAAV-TRE2G::DIO-ChR2(T159C)-mCherry plasmid was constructed by replacing the human elongation factor 1 α (EF1α) promotor sequence with the second generation tetracycline-responsive element (TRE2G) promotor sequence derived from the pLenti6PW-TGB plasmid [12, 34]. The EF1α promoter sequence was removed from the pAAV-EF1α::DIO-ChR2(T159C)-mCherry plasmid using MluI and KpnI sites. The TRE2G promotor sequence was prepared from the pLenti6PW-TGB plasmid using EcoRI and BstXI sites. Obtained TRE2G sequence and adeno-associated virus (AAV) backbone with DIO-ChR2(T159C)-mCherry sequences were blunted with the Klenow fragment of *Escherichia coli* DNA polymerase I. The TRE2G sequence was then subcloned into AAV backbone with DIO-ChR2(T159C)-mCherry sequences, generating the pAAV-TRE2G::DIO-ChR2(T159C)-mCherry plasmid.

The recombinant AAV vectors were then produced as described previously [35, 36]. Mice were injected with AAV9-TRE2G::DIO-ChR2(T159C)-mCherry at a titer of 1.3 × 10^13^ viral genomes (vg)/ml or AAV9-EF1α::DIO-ChR2(T159C)-mCherry at a titer of 1.8 × 10^12^ to 5.4 × 10^12^ vg/ml.

### Stereotactic surgery and cannula placement

Surgeries were carried out as described previously [12, 13, 17, 37]. The mice were anesthetized with isoflurane, given intraperitoneal injections of medetomidine hydrochloride (0.75 mg/kg of body weight), midazolam (4 mg/kg of body weight), and butorphanol tartrate (5 mg/kg of body weight) and then placed in a stereotactic apparatus (Narishige, Tokyo, Japan). For the optical stimulation experiments, 0.3 μl of AAV9-TRE2G::DIO-ChR2(T159C)-mCherry was bilaterally injected into the CA3 region (anterior-posterior [AP], − 2.0 mm; medial-lateral [ML], ±2.3 mm from bregma; doral-ventral [DV], − 2.0 mm from dura) using a glass micropipette filled with mineral oil attached to a 10-μl Hamilton microsyringe. A microsyringe pump (Narishige, Tokyo, Japan) and its controller were used to control the speed of the injection (0.1 μl/min). The needle was slowly lowered to the target site and remained there for 3 min after the injection. Then, stainless steel guide cannulas (internal diameter, 0.29 mm; outer diameter, 0.46 mm; Plastics One, Roanoke, VA) were bilaterally implanted in the CA3 areas (AP, − 2.0 mm; ML, ±2.3 mm; DV, − 1.0 mm from bregma). Microscrews were anchored in the skull near bregma and lambda, and the guide cannulas were fixed in place with dental cement. After the surgery, dummy cannulas with caps were inserted into the guide cannulas as protective covers. Mice were 11–18 weeks old at the time of surgery, and were allowed to recover 16–19 weeks before being used in behavioral experiments.

To establish the labeling system (for ChR2-mCherry-positive cell counting), the same surgical procedure was used except that the cannulas were not implanted. For this, the mice were 17–27 weeks old at the time of surgery and allowed to recover 5–10 weeks before being used in behavioral experiments.

For in vivo recording experiments, 0.3 μl of AAV9-EF1α::DIO-ChR2(T159C)-mCherry was injected into the CA3 regions of the right hemispheres (AP, − 2.0 mm; ML, + 2.3 mm from bregma; DV, − 2.0 mm from dura) using a glass micropipette. The mice were 13–18 weeks old at the time of surgery and were allowed to recover 21–41 weeks before being used in in vivo recording experiments.

### Behavioral analyses and optical stimulation

The home cage of each mouse was placed on a desk in the animal housing room for approximately 10 min before being transferred to the adjoining experimental room. For behavioral tests, each mouse was gently caught at the base of its tail and transferred to each context described below.

Context A was a cylindrical chamber (diameter, 180 mm; height, 230 mm) with a white acrylic floor and walls covered with black tape (see Additional file 1). Context B was a square-type chamber (175 × 165 mm; height, 300 mm) with a transparent acrylic board front, white sides and back walls, and a floor consisting of 26 stainless steel rods with a diameter of 2 mm placed 5 mm apart with a scented tray containing 0.25% benzaldehyde underneath (Additional file 1). The rods were connected to a shock generator via a cable harness. Constant minimal illumination was provided by a small light in the chamber. Context C was a square-type chamber (290 × 250 mm; height, 290 mm) with a transparent acrylic board front wall partially covered with white tape, grey side and back walls, and a floor consisting of grey acrylic board covered with white Kimtowels (Additional file 1). The room lights were off for context B but on for contexts A and C.

The c-fos::tTA/KA1::Cre double transgenic mice were 11–18 weeks old and 17–27 weeks old at the time of surgery for the optical stimulation experiment and for the establishment of the labeling system (ChR2-mCherry-positive cells counting), respectively. Cannula-implanted and AAV-injected c-fos::tTA/KA1::Cre double transgenic mice were maintained on 40 mg/kg Dox food pellets in a microisolation rack system (FRP BIO2000, CLEA Japan) consisting of 16 individually ventilated boxes (1–4 cages/box) with glass fiber filters. Mice were used for behavioral analysis 16–19 weeks and 5–10 weeks after surgery for the optical stimulation experiment and for the establishment of the labeling system (ChR2-mCherry positive cells counting), respectively. Before starting behavioral experiment, the mice were kept under the condition of Dox withdrawal (OFF Dox) for 2 days and then exposed to context A for 6 min. One day later, the mice were subjected to contextual fear conditioning (CFC) in context B, consisting of 3 unsignaled foot shocks (2-s duration, 0.4 mA, 1 min apart) beginning 2 min after acclimation. After the last shock, the mice remained in the context for 1 min and were then returned to their home cages. One day later, the mice were anesthetized with approximately 2.0% isoflurane, and the dummy cannulas were replaced with two-branch-type optical fiber units comprising a plastic cannula body and a tightly connected 0.25-mm-diameter optic fiber (COME2-DF2–250; Lucir, Ibaraki, Japan). The tip of the optical fiber was targeted slightly above CA3 (DV, − 1.5 mm from bregma). The mice were then returned to their home cages for 1–1.5 h. For the optical stimulation session, the mice were moved to the experimental room, and the fiber unit was connected to an optical swivel (COME2-UFC; Lucir), which was connected to a laser (200 mW, 473 nm, COME-LB473/200; Lucir) via a main optical fiber. The delivery of laser pulses was controlled by a schedule stimulator (COME2-SPG-2; Lucir) operating in time-lapse mode. The mice in their home cages were subjected to ten trains of laser pulses, each consisting of 300, 500-μs pulses at 20-Hz of 473 nm light (approximately 10 mW output from the fiber tip) with 45-s intertrain intervals. Approximately 1–1.5 h after the laser stimulation, the mice were anesthetized with approximately 2.0% isoflurane, the optic fiber unit was detached, and the mice were again returned to their home cages. The mice were then given food containing 1000 mg/kg Dox for 2 days and then maintained on food containing 40 mg/kg Dox.

To test fear memory, the mice were placed in contexts A, B, and C for 3 min each at 1, 2, and 3 days after the optical stimulating session, respectively. At the end of each session, the mice were returned to their home cages and the contexts were cleaned with water and 80% ethanol. A video tracking system (Muromachi Kikai, Tokyo, Japan) was used to measure the freezing behavior of the animals, as described in previous studies [12, 13, 17, 38, 39]. Freezing was defined as no movement detected for > 1.5 s. All training and testing were conducted during the light phase of the light-dark cycle. The mean values of the freezing responses during each session were analyzed except for the context A pre-exposure session, for which freezing responses during first 3 min were analyzed.

For the animals used for ChR2-mCherry-positive cell counting, the behavioral experiments were conducted as described above until CFC in context B. One day after CFC, the mice were perfused and histologically analyzed.

### In vivo recordings

In vivo recordings were carried out as described previously [17, 38–42], with modification for the optogenetic stimulation. Mice previously infected with AAV9-EF1α::DIO-ChR2(T159C)-mCherry were anesthetized with urethane (1.2 g/kg) and placed in a stereotactic apparatus. An optic fiber (COME2-DF1–250; Lucir) was glued to the recording tungsten electrode (WE 40 mm 0030.1B5, 100 kΩ; MicroProbes, Gaithersburg, MD) so that the tip of the fiber was 500 μm above the tip of the electrode. The optrode was slowly inserted into the CA3 region of the right hemisphere, and the optic fiber unit was connected to an optical swivel (COME2-UFC) connected to a laser (COME-LB473/200). The body temperatures of the mice were maintained by keeping them on a heating pad (MK-900; Muromachi Kikai, or ATC-TY; Unique-Medical Inc., Tokyo, Japan) during the recording sessions.

The delivery of laser pulses was controlled by a Master 8 device (A.M.P. Instruments, Jerusalem, Israel). Optically evoked field excitatory postsynaptic potentials (fEPSPs) was recorded by using optical test pulses (stimulation frequency, 0.033-Hz, 500-μs pulses). After establishing a stable baseline at the recording site for 30 min, a 20-Hz optical stimulating protocol was conducted, which was followed by 0.033-Hz test pulses for 3 h. The 20-Hz optical stimulating protocol was identical to that used in the behavioral analysis, except the laser power was the same as that used for the optical test pulses. The stimulation laser power was adjusted to approximately half of the maximum fEPSP amplitude.

Signals were amplified and filtered from 5-Hz to 1-kHz with a Bioelectric amplifier (MEG-1200; Nihon Kohden, Tokyo, Japan), digitized by Digidata (1322A or 1550B; Axon Instruments, Molecular Devices, San Jose, CA), and sampled at 10-kHz using Clampex software (version 9.2 or 10.7). The data were analyzed with Clampfit 10.7 software. All animals were perfused after the recordings, and the positions of the recording sites were verified.

### Histology

The mice were deeply anesthetized with an overdose of pentobarbital solution and perfused transcardially with 4% paraformaldehyde in phosphate-buffered saline (PBS; pH 7.4). The brains were removed and further post-fixed by immersion in 4% paraformaldehyde in PBS for 24 h at 4 °C. Each brain was equilibrated in 25% sucrose in PBS and then frozen in dry-ice powder. Coronal sections of 30 μm (for ChR2-mCherry-positive cell counting) or 50 μm thickness (after in vivo recording) were cut on a cryostat and transferred to 12-well cell culture plates (Corning, Corning, NY) containing PBS. After washing with PBS, the floating sections were treated with 4′,6-diamidino-2-phenylindole (DAPI) (1 μg/ml, 10,236,276,001; Roche Diagnostics) at room temperature for 20 min and then washed with PBS three times (3 min per wash). The sections were mounted on slide glass with ProLong Gold antifade reagent (Invitrogen of Thermo Fisher Scientific, Waltham, MA). Images were acquired on a fluorescence microscope (BZ9000; Keyence, Osaka, Japan) with a Plan-Apochromat 20× objective lens (Nikon, Tokyo, Japan) for ChR2-mCherry-positive cell counting or with a Plan-Apochromat 10× objective lens (Nikon) for verification of the recording site. To quantify the number of ChR2-mCherry-positive cells, images of CA3 were acquired by collecting z-stacks (2.4 μm apart, 5–6 images). Maximum intensity projections of the images were created with the image analysis software (BZ-II; Keyence). Two sections (AP, approximately − 1.9 and − 2.0 mm from bregma) corresponding to each region of interest (ROI) (CA3 pyramidal cell layers from both hemispheres within 540 × 720 μm^2^) were chosen from each mouse, and the ChR2-mCherry-positive cells in the ROI were counted manually. The average number of ChR2-mCherry-positive cells per section from one hemisphere are presented throughout the text.

### Statistical analysis

Statistical analyses were performed using GraphPad Prism 6 (GraphPad Software, Inc., La Jolla, CA). Comparisons of data between two groups were analyzed with Student’s *t* tests (two tailed) or Welch’s *t* test (two tailed, without assuming homogeneity of variances), and multiple-group comparisons were assessed using one-way analyses of variance (ANOVAs) followed by Tukey’s post hoc multiple-comparisons tests when significant main effects were detected. In Fig. 3e, data were analyzed with two-way repeated measures (RM) ANOVA. When significant main effects were detected, Bonferroni’s multiple comparisons test were used for post hoc tests. Quantitative data are expressed as the means ± standard errors of the means (SEMs).

## Results

### A system for manipulation of cell ensembles in CA3

**Fig. 1:**
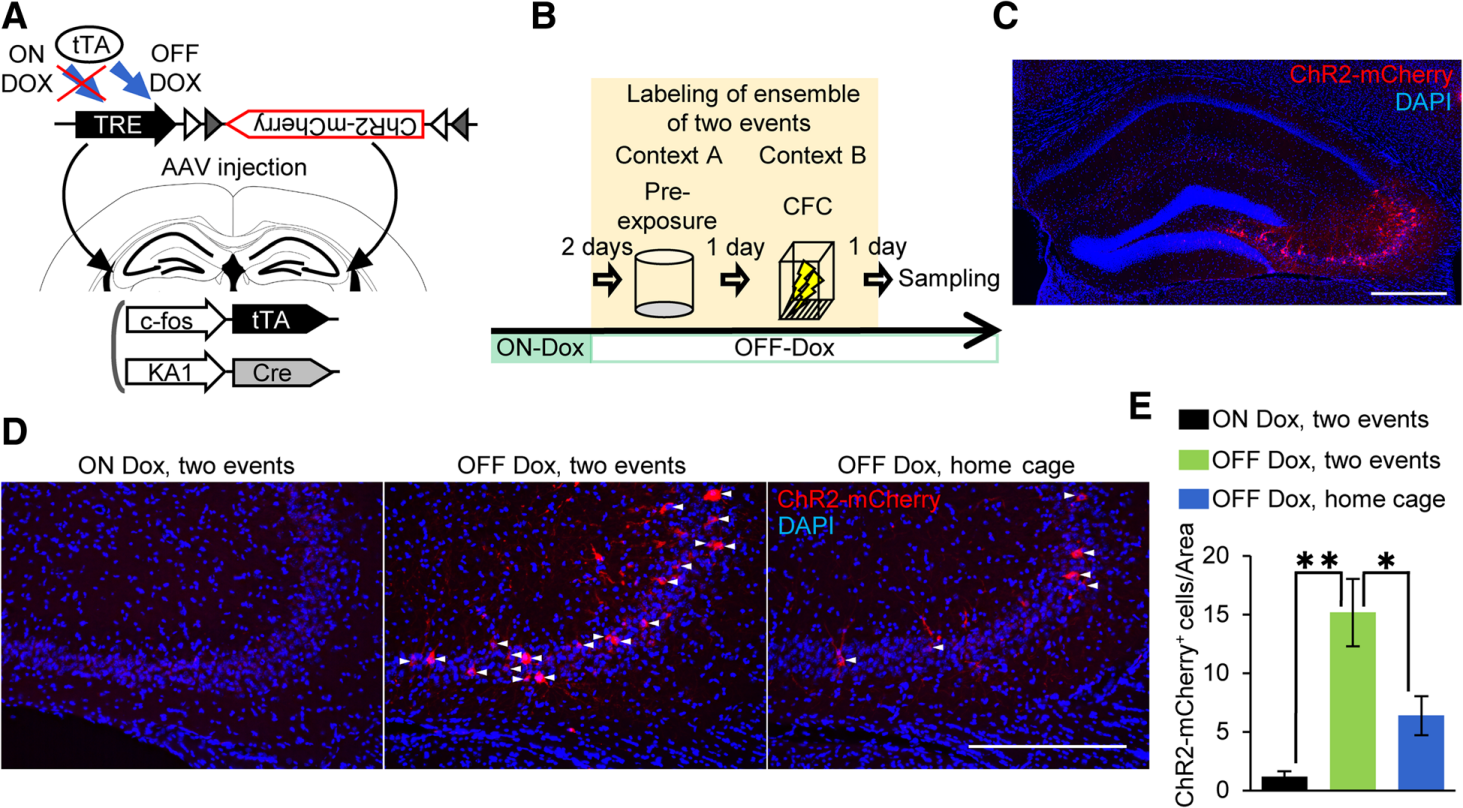
System for activity-dependent labeling of CA3 neurons. **a** Diagram showing the activity-dependent labeling of neurons in c-fos:: tTA/KA1::Cre double transgenic mice with an AAV system. Gray and white triangles represent loxP and lox2272 sequences, respectively. **b** Scheme of the experiment. Neural activity was elicited during context A pre-exposure and CFC in context B (i.e., the two events) in animals off of doxycycline treatment (OFF Dox). **c** Representative image of ChR2-mCherry expression in the CA3 region of the hippocampus of an AAV-injected c-fos::tTA/KA1::Cre double transgenic mouse exposed to the two events in the OFF Dox condition. Scale bar, 500 μm. **d** Representative images of ChR2-mCherry expression in the CA3 regions of AAV-injected c-fos::tTA/KA1::Cre double transgenic mice exposed to the two events in an ON Dox condition (left) or an OFF Dox condition (middle) and in mice in the home cage in the OFF Dox condition (right). The ChR2-mCherry signal is in red and DAPI nuclear staining is in blue. Arrowheads indicate ChR2-mCherry-positive cells. Scale bar, 300 μm. **e** Graph showing the average numbers of ChR2-mCherry-positive cells per section in the CA3 region from one hemisphere (counted from both hemisphere, two sections from 4 mice/group, one-way ANOVA; F_2, 9_ = 13.4, *p* = 0.0020; Tukey’s post hoc multiple-comparisons test, *p < 0.05, **p < 0.01)

We used c-fos::tTA/KA1::Cre double transgenic mice infected with AAV vectors harboring a light-activated cation channel, ChR2 [43], to genetically label CA3 cell ensembles corresponding to specific events (Fig. 1a). In these mice, the expression of ChR2 from the viral vector is driven by the immediate early gene c-fos, which is induced by neural activity. In the absence of Dox, tTA binds to the TRE to drive the expression of the target gene specifically in the CA3 region of the hippocampus via the Cre-loxP system from the KA1::Cre mice [29]. Therefore, the subpopulation of cells in CA3 activated during learning will express ChR2-mCherry (Fig. 1b, c, and d). Specifically, neural activity was elicited during context A pre-exposure and CFC in context B (referred to as “two events”) when the animals were off Dox. ChR2-mCherry-positive cells were only observed in OFF Dox animals, and the number of ChR2-mCherry-positive cells were significantly increased by the two events (Fig. 1d and e; 4 mice/group, one-way ANOVA; F_2,9_ = 13.4, *p* = 0.0020; Tukey’s post hoc multiple-comparisons test, OFF Dox, two events vs. ON Dox, two events, *p* = 0.0016). For the OFF Dox condition, the labeling efficiency in mice kept in their home cages was significantly lower than in those exposed to the two events (Tukey’s post hoc multiple-comparisons test, OFF Dox, two events vs. OFF Dox, home cage, *p* = 0.0256). These data show that the cell ensembles in CA3 corresponding to the two events are specifically labeled with this system.

### Synchronous activation of cell ensembles in CA3 associates distinct events

**Fig. 2:**
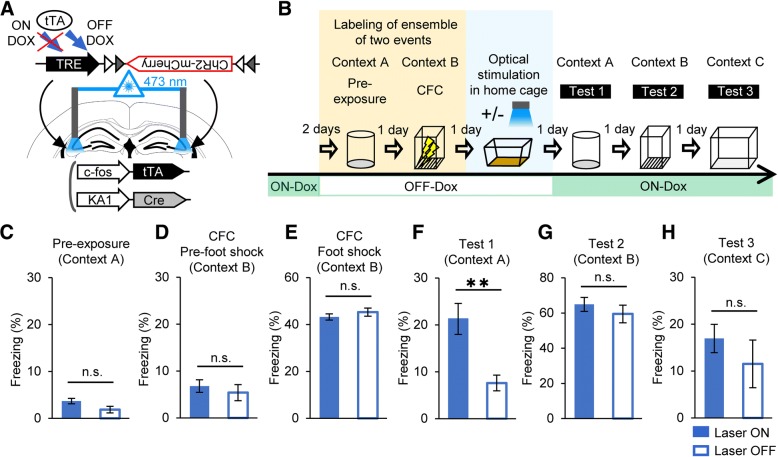
Optogenetic stimulation of cell ensembles in CA3 associates distinct events. **a** Diagram showing the cannula placement and activity-dependent labeling of neurons in c-fos::tTA/KA1::Cre double transgenic mice with the AAV system. Gray and white triangles represent loxP and lox2272 sequences, respectively. **b** Diagram showing the experimental scheme. After bilateral CA3 infection with the AAV, the c-fos::tTA/KA1::Cre double transgenic mice were exposed to context A and CFC in context B in OFF Dox condition. One day after CFC, the CA3 regions of the laser ON group (*n* = 12) were optically stimulated, whereas the laser OFF group (*n* = 10) received no optical stimulation. Mice were tested for their freezing responses in contexts A, B, and C on 1, 2, and 3 days after the optical stimulation, respectively. Graphs showing freezing responses during pre-exposure to context A (unpaired Student’s *t* test, *t*_20_ = 1.255, *p* = 0.2239) (**c**), pre-foot shock session during CFC in context B (unpaired Student’s *t* test, *t*_20_ = 0.6455, *p* = 0.5259) (**d**), and during and after foot shock session of CFC in context B (Welch’s *t* test; *t*_12.62_ = 0.2795, *p* = 0.7844) (**e**). Results of the tests in context A (Welch’s *t* test; *t*_16.22_ = 3.809, *p* = 0.0015) (**f**), context B (unpaired Student’s *t* test; *t*_20_ = 0.8214, *p* = 0.4211) (**g**), and the novel context C (unpaired Student’s *t* test; *t*_20_ = 0.8279, *p* = 0.4175) (**h**). **p < 0.01; n.s., no significant difference between the two groups; error bars are the means ± SEMs

The CA3-specific labeling system was used to examine whether the simultaneous activation of cell ensembles in this region corresponding to each of the two events (i.e., the context A pre-exposure and CFC in context B) generates an artificial association between them. For this, the c-fos::tTA/KA1::Cre double transgenic mice harboring the ChR2-mCherry viral vector and guide cannulas in bilateral CA3 regions (Fig. 2a) were divided into two groups, a laser ON group (*n* = 12) subjected to optical stimulation and a laser OFF group (*n* = 10), which received no laser stimulation as a control. After AAV injections and cannula implantations, the mice were taken off Dox to enable neural activity-dependent labeling of cells and then subjected to the two events as described above (Fig. 2b). During these sessions, there were no significant differences in freezing between the laser ON and laser OFF groups (Fig. 2c–e; context A pre-exposure session, unpaired Student’s *t* test; *t*_20_ = 1.255, *p* = 0.2239; pre-foot shock session during CFC, unpaired Student’s *t* test; *t*_20_ = 0.6455, *p* = 0.5259; during and after foot shock session of CFC, Welch’s *t* test; *t*_12.62_ = 0.2795, *p* = 0.7844). One day after CFC, the laser ON group was subjected to bilateral 20-Hz laser stimulation of CA3 in their home cages to synchronously activate all cell ensembles. Mice in the laser OFF group were attached to optic fibers targeting CA3, but the laser pulses were not delivered.

Beginning the day after the optical stimulation, the mice were subjected to fear memory tests over three consecutive days using the different contexts. When mice were tested in context A, in which they had not experienced CFC, the laser ON group showed significantly more freezing than the laser OFF group (Fig. 2f; Welch’s *t* test, *t*_16.22_ = 3.809, *p* = 0.0015). In context B, in which they were subjected to CFC, there was no difference in freezing between the two groups (Fig. 2g; unpaired Student’s *t* test; *t*_20_ = 0.8214, *p* = 0.4211). Furthermore, there was no significant difference in freezing in the novel context C (Fig. 2h, unpaired Student’s *t* test; *t*_20_ = 0.8279, *p* = 0.4175), demonstrating that the observed fear memory was context specific. These results indicate that two distinct events can be associated via the synchronous activation of the corresponding cell ensembles in CA3.

### Optogenetic stimulation induces LTP within the CA3-CA3 recurrent circuit

**Fig. 3:**
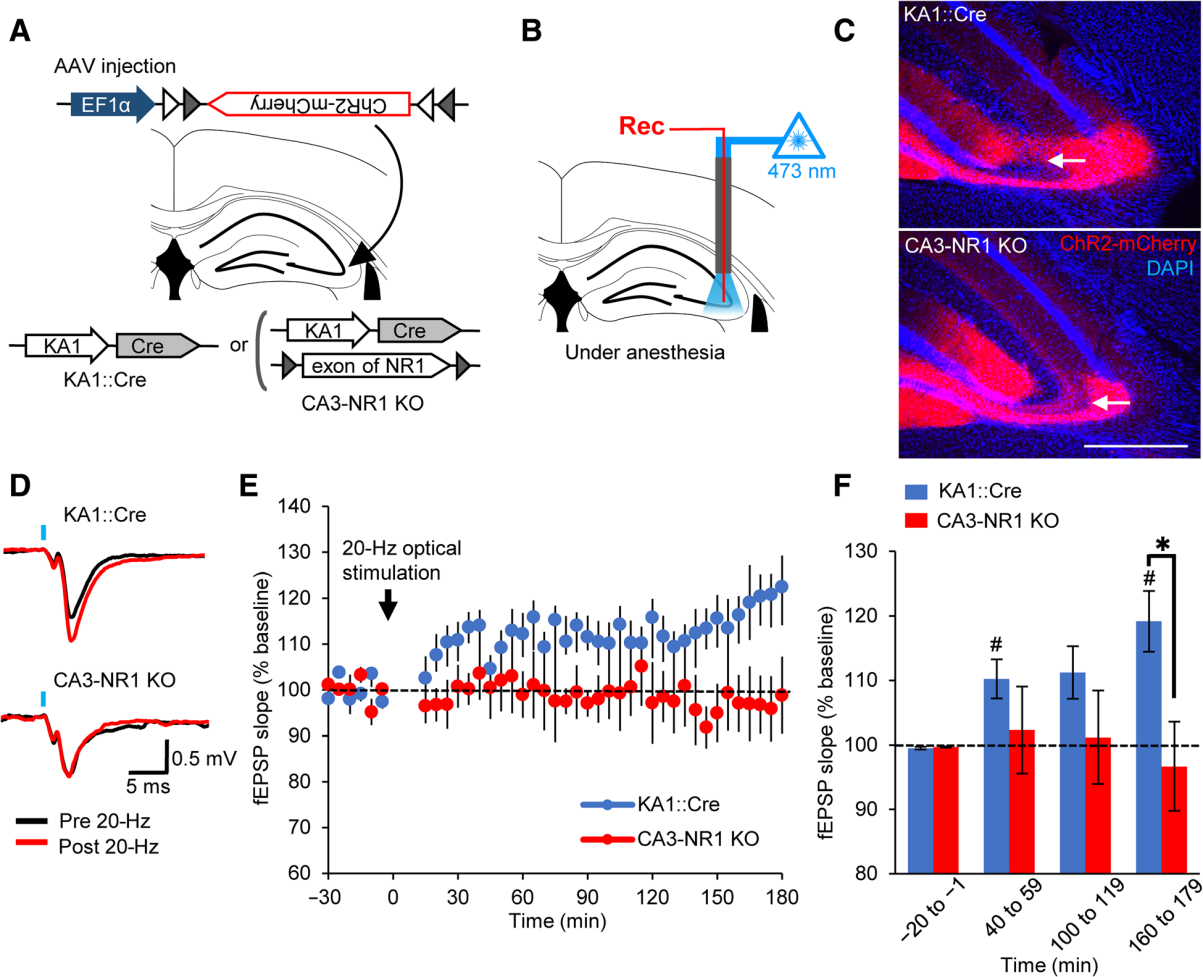
Optogenetic stimulation of CA3 induces LTP within CA3-CA3 recurrent synapses. **a** KA1::Cre or CA3-NR1 KO mice received injections of AAV into the CA3 regions of their right hemispheres. Gray and white triangles represent loxP and lox2272 sequences, respectively. **b** After AAV infection, field excitatory postsynaptic potentials (fEPSPs) were recorded while the mice were under urethane anesthesia. Rec, recording electrode. **c** Representative images of ChR2-mCherry expression in the CA3 regions of KA1::Cre and CA3-NR1 KO mice. Arrows indicate the tips of the recording electrodes. Scale bar, 500 μm. The loci of the recording electrodes were confirmed by histology (see Additional file 2). **d** Representative traces showing LTP in the CA3 in the KA1::Cre mice but not in the CA3-NR1 KO mice after the 20-Hz optical stimulation. Black and red traces represent baseline and 160 min after 20-Hz stimulation periods, respectively. Blue columns indicate the time point of the optical test pulse (473 nm, 500 μs). **e** Plot showing the time course of fEPSP slopes after 20-Hz optical stimulation (*n* = 4 mice/group, Two-way RM ANOVA; interaction, F_42,129_ = 1.418, *p* = 0.0709; time, F_42,129_ = 0.6796, *p* = 0.9248, genotype; F_1,129_ = 160.7, *p* < 0.0001; Bonferroni’s multiple comparisons test, KA1::Cre vs. CA3-NR1 KO, *p* < 0.05, 155–175 min after the 20-Hz laser stimulation). **f** Mean values of fEPSP slopes between − 20 and − 1 min, 40 and 59 min, 100 and 119 min, and 160 and 179 min (*n* = 4 mice/group; KA1::Cre: − 20 to − 1 min vs. 40–59 min, paired Student’s *t* test; *t*_3_ = 3.440, *p* = 0.0412; KA1::Cre: − 20 to − 1 min vs. 160–179 min, paired Student’s *t* test; *t*_3_ = 4.327, *p* = 0.0228; CA3-NR1 KO: − 20 to − 1 min vs. 40–59 min, paired Student’s *t* test; *t*_3_ = 0.3868, *p* = 0.7247; CA3-NR1 KO: − 20 to − 1 min vs. 160–179 min, paired Student’s *t* test; *t*_3_ = 0.4318, *p* = 0.6951; 160–179 min: KA1::Cre vs. CA3-NR1 KO, unpaired Student’s *t* test; *t*_6_ = 2.690, *p* = 0.036). Significant difference between genotype and baseline at */#*p* < 0.05, error bars are the means ± SEMs

To study whether the 20-Hz optogenetic stimulation that induced the artificial association described above also induces LTP at CA3-CA3 synapses, in vivo extracellular recordings were performed in the CA3 regions of anesthetized KA1::Cre mice or CA3-NR1 KO mice [29] infected AAV-EF1α::DIO-ChR2-mCherry in CA3 (Fig. 3a, b). It is reported that LTP at CA3-CA3 recurrent synapses depends on NMDA receptor of the CA3 whereas dysfunction of NMDA receptors in the CA3 has no effect on the LTP between mossy fiber-CA3 synapses or Shaffer collateral-CA1 synapses [29, 44–46]. Therefore, CA3-NR1 KO mice was used to verify that the observed LTP in KA1::Cre mice is attributed to LTP within CA3-CA3 synapses.

ChR2-mCherry expression was observed in the CA3 regions of AAV-infected animals (Fig. 3c); the loci of recording electrodes were identified histologically after completion of the recordings (Additional file 2). During 20-Hz laser stimulation, field responses followed stimulations faithfully in both the KA1::Cre mice and the CA3-NR1 KO mice (Additional file 3). There was no statistical difference of input-output function between the groups (Additional file 4; 50% stimulation intensity, unpaired Student’s *t* test; *t*_6_ = 0.6807, *p* = 0.5214; 100% stimulation intensity, unpaired Student’s *t* test; *t*_6_ = 0.8144, *p* = 0.4465; 50% fEPSP amplitude, unpaired Student’s *t* test; *t*_6_ = 0.6289, *p* = 0.5526; 100% fEPSP amplitude, *t*_6_ = 0.7006, *p* = 0.5098).

A progressive increase in the fEPSP slopes was recorded in KA1::Cre mice (Fig. 3d–f; *n* = 4 mice/group, − 20 to − 1 min vs. 40–59 min, paired Student’s *t* test; *t*_3_ = 3.440, *p* = 0.0412; − 20 to − 1 min vs. 160 to 179 min, paired Student’s *t* test; *t*_3_ = 4.327, *p* = 0.0228) but not in CA3-NR1 KO mice (Fig. 3d–f; − 20 to − 1 min vs. 40–59 min, paired Student’s *t* test; *t*_3_ = 0.3868, *p* = 0.7247; − 20 to − 1 min vs. 160 to 179 min, paired Student’s *t* test; *t*_3_ = 0.4318, *p* = 0.6951). The fEPSP slope recorded in KA1::Cre mice was significantly higher than in CA3-NR1 KO mice after 20-Hz laser stimulation (Fig. 3e; Two-way RM ANOVA; interaction, F_42,129_ = 1.418, *p* = 0.0709; time, F_42,129_ = 0.6796, *p* = 0.9248, genotype; F_1,129_ = 160.7, *p* < 0.0001). This difference was found 160–175 min after the 20-Hz laser stimulation (Fig. 3e, f; Bonferroni’s multiple comparisons test, KA1::Cre vs. CA3-NR1 KO, *p* < 0.05, 155–175 min after the 20-Hz laser stimulation; 160–179 min, unpaired Student’s *t* test; *t*_6_ = 2.690, *p* = 0.036). These results indicate that 20-Hz laser stimulation of ChR2-expressing neurons of the CA3 induces LTP within CA3-CA3 synapses.

## Discussion

Pattern completion, a retrieval process of associative memory, is a well-known function of the recurrent network in CA3 [27–29]. Moreover, the mechanism avoiding interference between pre-stored information within CA3-CA3 recurrent synapses and new information have been advocated [47, 48]. Previous gain-of-function studies using an optogenetic technique showed that manipulation of the DG [4, 8, 21–24] or CA1 [12] engrams is important for memory reactivation and to generate synthetic or false memory by linking between stored information and sensory input by artificial activation of cell ensembles. However, CA3 gain-of-function study has not been reported and it was not known whether CA3, which has recurrent circuit is involved in the generation of associations among the stored information. Here, we show that simultaneous optogenetic activation of CA3 cell ensembles bridges two initially independent events.

Ohkawa et al. [12] induced association between pre-stored information within CA1 and basolateral amygdala, whereas our study induced association between pre-stored information within one brain region, CA3. Our results imply that CA3 composing recurrent circuit has unique characteristics to bridge the memories and to generate new memories among the multiple memories. To the best of our knowledge, this study is the first gain-of-function study in CA3 demonstrating that the association between individual units of stored information occurs within a single brain region, namely, the CA3 of the hippocampus.

LTP within CA3-CA3 recurrent synapses depends on NMDA receptor of CA3 whereas dysfunction of NMDA receptors in the CA3 has no effect on the LTP between mossy fiber-CA3 synapses or Shaffer collateral-CA1 synapses [29, 44–46]. Our study showed that, 20-Hz optogenetic stimulation induced LTP within the CA3 whereas this LTP was abolished in CA3-NR1 KO mice (Fig. 3d–f). These data indicate that, our system detects the LTP within CA3-CA3 recurrent synapses.

The subpopulation of CA3 neurons activated during contextual learning are reactivated specifically during exposure to the learned context and during memory retrieval [2]. Thus, distinct CA3 cell ensembles may be activated in distinct contexts, such as during the two events (pre-exposure and CFC) in this study. These two initially separate “memories” can be associated to generate new memories by synchronous activation of the corresponding cell ensembles. The results of our electrophysiology showed that optogenetic stimulation of CA3 induce LTP within CA3 recurrent synapses. It is reported that, LTP at appropriate synapses are both necessary and sufficient for information storage [16, 17]. Moreover, LTP also contributes to associative learning or memory update [18–20]. A potential mechanism underlying memory association observed in our study is that optogenetic stimulation increased synaptic efficacy between cell ensembles within the CA3 recurrent circuit, generating new functional connection. This newly generated connection leads to the activation of the cell ensemble corresponding to the pre-exposure followed by the activation of the cell ensemble corresponding to CFC. Thus, recall of the pre-exposure memory may triggers the recall of the CFC memory and induces freezing behavior. The sharing of a memory ensemble may also emerge in regions downstream of CA3 to associate the events [12, 13, 37, 49, 50].

The results of this study show that stimulation at 20-Hz, a frequency lower than that used for theta burst stimulation, is sufficient to induce LTP in the CA3. A recent study reported that CA3-CA3 synapses exhibit a unique “symmetrical” spike timing-dependent plasticity curve, in which LTP is induced regardless of pre-post spike timing with a relatively large interval (half-width, ~ 150 ms) [28]. This spike timing-dependent plasticity may have contribute to the induction of LTP observed here with 20-Hz (50-ms interval) optogenetic stimulation. Further study is needed to determine the contribution of spike timing-dependent plasticity in the CA3 recurrent circuit to associative memory.

## Additional files

Additional file 1: Contexts used for the behavioral experiments. Photographs showing contexts A, B, and C used in this study. (PDF 2587 kb)

Additional file 2: Histological verification of electrode tips for in vivo electrophysiological recording. Placements of recording electrode tips (red circles) in KA1::Cre (left) and CA3-NR1 KO (right) mice. (PDF 3849 kb)

Additional file 3: In vivo optically evoked synaptic responses in CA3. Envelopes of field responses to 20-Hz optical stimulation, obtained from KA1::Cre (top) and CA3-NR1 KO (bottom) animals infected with AAV-EF1α::DIO-ChR2-mCherry in the CA3 region. Note that the responses follow the stimulation faithfully. Blue columns indicate the timing of laser stimulation. (PDF 1486 kb)

Additional file 4: Input-output function. Data of input-output function obtained from KA1::Cre and CA3-NR1 KO mice infected with AAV-EF1α::DIO-ChR2-mCherry in the CA3 region. Laser power and fEPSP amplitude at the time of getting maximum fEPSP amplitude and at the time of using 50% of this stimulation intensity are shown. There was no statistical difference of input-output function between the groups (50% stimulation intensity, unpaired Student’s *t* test; *t*_6_ = 0.6807, *p* = 0.5214; 100% stimulation intensity, unpaired Student’s *t* test; *t*_6_ = 0.8144, *p* = 0.4465; 50% fEPSP amplitude, unpaired Student’s *t* test; *t*_6_ = 0.6289, *p* = 0.5526; 100% fEPSP amplitude, *t*_6_ = 0.7006, *p* = 0.5098). (XLS 27 kb)

## Abbreviations

AAV: Adeno-associated virus
ANOVA: Analysis of variance
CFC: Contextual fear conditioning
ChR2: Channelrhodopsin-2
DAPI: 4′,6-diamidino-2-phenylindole
Dox: Doxycycline
EF1α: Human elongation factor 1 α
fEPSP: Field excitatory postsynaptic potential
KO: Knock-out
LTP: Long-term potentiation
NMDA: *N*-methyl-D-aspartate
NR1: NMDA receptor subunit 1
PBS: Phosphate-buffered saline
PCR: Polymerase chain reaction
SEM: Standard error of mean
TRE2G: Second-generation tetracycline-responsive element
tTA: Tetracycline transactivator
