# Supplementary figures and images for: Artificial association of memory events by optogenetic stimulation of hippocampal CA3 cell ensembles
Source: Mol Brain. 2019 Jan 8;12:2. doi: 10.1186/s13041-018-0424-1 (PMC6323779; doi:10.1186/s13041-018-0424-1)

Context A

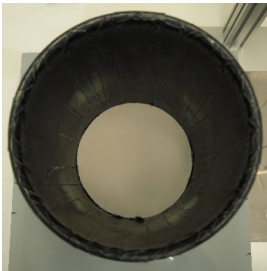

Context B

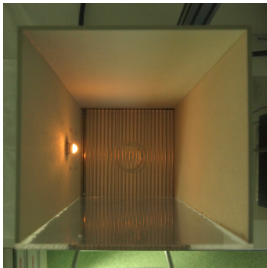

Context C

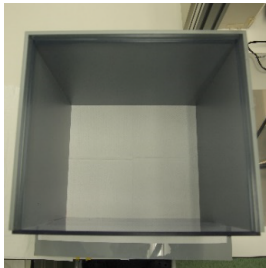

Supplement: Supplementary file 1 — Contexts used for the behavioral experiments. Photographs showing contexts A, B, and C used in this study. (PDF 2587 kb) [file 13041_2018_424_MOESM1_ESM.pdf]

KA1::Cre

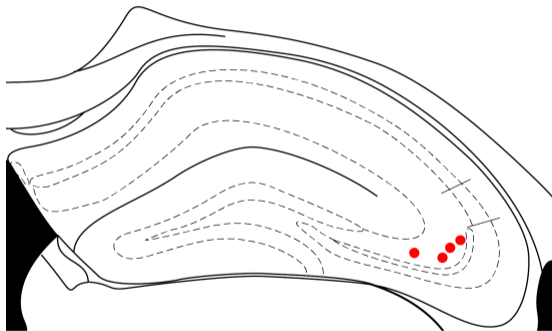

CA3-NR1 KO

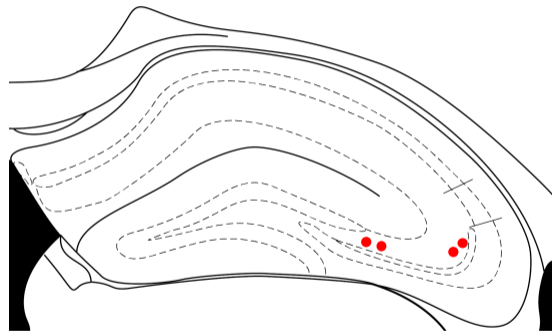

AP -2.0 mm

Supplement: Supplementary file 2 — Histological verification of electrode tips for in vivo electrophysiological recording. Placements of recording electrode tips (red circles) in KA1::Cre (left) and CA3-NR1 KO (right) mice. (PDF 3849 kb) [file 13041_2018_424_MOESM2_ESM.pdf]

KA1::Cre

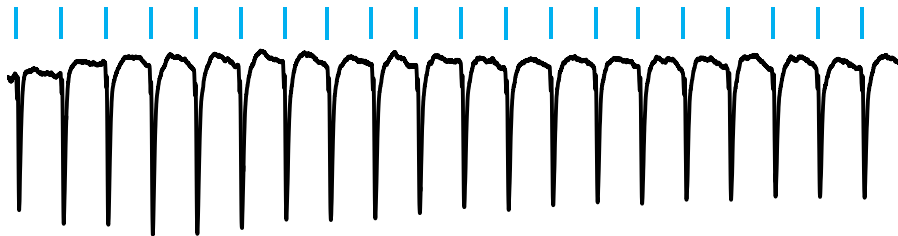

CA3-NR1 KO

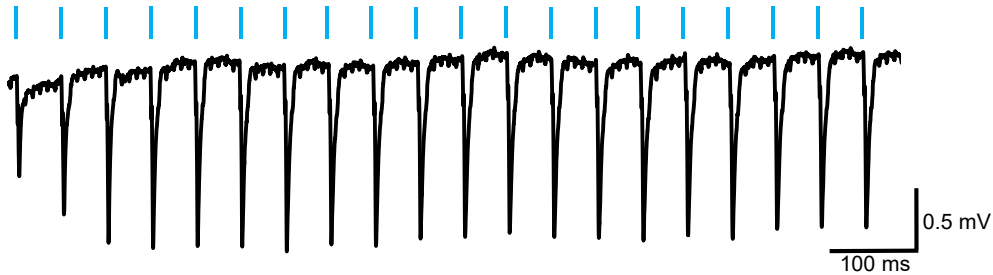

Supplement: Supplementary file 3 — In vivo optically evoked synaptic responses in CA3. Envelopes of field responses to 20-Hz optical stimulation, obtained from KA1::Cre (top) and CA3-NR1 KO (bottom) animals infected with AAV-EF1α::DIO-ChR2-mCherry in the CA3 region. Note that the responses follow the stimulation faithfully. Blue columns indicate the timing of laser stimulation. (PDF 1486 kb) [file 13041_2018_424_MOESM3_ESM.pdf]
